# Supplementary material for: In Search of Species-Specific SNPs in a Non-Model Animal (European Bison (Bison bonasus))—Comparison of De Novo and Reference-Based Integrated Pipeline of STACKS Using Genotyping-by-Sequencing (GBS) Data
Source: Animals (Basel). 2021 Jul 28;11(8):2226. doi: 10.3390/ani11082226 (PMC8388393; doi:10.3390/ani11082226)
Supplement: Supplementary file 1 [file animals-11-02226-s001.zip › animals-1274303-supplementary.pdf]

Table S1. Total number of reads per run before de multiplexing

S.No.	SAMPLE RUN	READS
1	181101_I133_FCHTGWFBBXX_L8_EURtsgG1AAD32FAAPEI-37_1.fq.gz	103331102
2	181101_I133_FCHTGWFBBXX_L8_EURtsgG1AAD32FAAPEI-37_2.fq.gz	103331102
3	181101_I133_FCHTGWFBBXX_L8_EURtsgG1ABD32FAAPEI-39_1.fq.gz	101160593
4	181101_I133_FCHTGWFBBXX_L8_EURtsgG1ABD32FAAPEI-39_2.fq.gz	101160593
5	181108_I133_FCH2NJCBBXY_L6_EURtsgG1ACD32FAAPEI-40_1.fq.gz	48339898
6	181108_I133_FCH2NJCBBXY_L6_EURtsgG1ACD32FAAPEI-40_2.fq.gz	48339898
7	181108_I133_FCH2NJCBBXY_L7_EURtsgG1ACD32FAAPEI-40_1.fq.gz	49148511
8	181108_I133_FCH2NJCBBXY_L7_EURtsgG1ACD32FAAPEI-40_2.fq.gz	49148511
9	181108_I133_FCH2NJCBBXY_L6_EURtsgG1ADD40FAAPEI-41_1.fq.gz	74616236
10	181108_I133_FCH2NJCBBXY_L6_EURtsgG1ADD40FAAPEI-41_2.fq.gz	74616236
11	181108_I133_FCH2NJCBBXY_L7_EURtsgG1ADD40FAAPEI-41_1.fq.gz	75614886
12	181108_I133_FCH2NJCBBXY_L7_EURtsgG1ADD40FAAPEI-41_2.fq.gz	75614886
13	190220_I137_FCH2MNKBBXY_L6_EURfjrG1ABD40FAAPEI-18_1.fq.gz	123795099
14	190220_I137_FCH2MNKBBXY_L6_EURfjrG1ABD40FAAPEI-18_2.fq.gz	123795099
15	190220_I137_FCH2MNKBBXY_L5_EURfjrG1AAD40FAAPEI-17_1.fq.gz	131099277
16	190220_I137_FCH2MNKBBXY_L5_EURfjrG1AAD40FAAPEI-17_2.fq.gz	131099277
17	190220_I137_FCH2MNKBBXY_L7_EURfjrG1ACD40FAAPEI-19_1.fq.gz	92268720
18	190220_I137_FCH2MNKBBXY_L7_EURfjrG1ACD40FAAPEI-19_2.fq.gz	92268720
19	SUM	1598748644
20	Min	48339898
21	Max	131099277
22	Average	88819369.1
23	Stdev	28644142.1
24	Median	92268720

Table S2. Total number of reads per sample after de multiplexing

S. No.	Sample_Name	Read Count
1	816_M_control.2.fq.gz	6333140
2	816_M_control.1.fq.gz	6333140
3	1340_M_control.2.fq.gz	6284322
4	1340_M_control.1.fq.gz	6284322
5	1021_M_control.2.fq.gz	5348543
6	1021_M_control.1.fq.gz	5348543
7	1142_M_case.2.fq.gz	4917367
8	1142_M_case.1.fq.gz	4917367
9	1196_M_case.2.fq.gz	4439779
10	1196_M_case.1.fq.gz	4439779
11	1011_M_control.2.fq.gz	4406926
12	1011_M_control.1.fq.gz	4406926
13	1139_M_control.2.fq.gz	4260862
14	1139_M_control.1.fq.gz	4260862
15	33K_M_control.2.fq.gz	4258410
16	33K_M_control.1.fq.gz	4258410
17	11K_M_case.2.fq.gz	4180062
18	11K_M_case.1.fq.gz	4180062
19	120R_M_case.2.fq.gz	4139492
20	120R_M_case.1.fq.gz	4139492
21	326_M_case.2.fq.gz	4109938
22	326_M_case.1.fq.gz	4109938
23	1181_M_case.2.fq.gz	4074021
24	1181_M_case.1.fq.gz	4074021
25	88R_M_control.2.fq.gz	4036062
26	88R_M_control.1.fq.gz	4036062
27	803_M_case.2.fq.gz	3872623
28	803_M_case.1.fq.gz	3872623
29	820_M_case.2.fq.gz	3839579
30	820_M_case.1.fq.gz	3839579

31	17K_F_case.2.fq.gz	3829158
32	17K_F_case.1.fq.gz	3829158
33	813_M_control.2.fq.gz	3799087
34	813_M_control.1.fq.gz	3799087
35	1008_M_case.2.fq.gz	3787800
36	1008_M_case.1.fq.gz	3787800
37	1072_M_case.2.fq.gz	3765567
38	1072_M_case.1.fq.gz	3765567
39	68_M_case.2.fq.gz	3753277
40	68_M_case.1.fq.gz	3753277
41	819_M_case.2.fq.gz	3729276
42	819_M_case.1.fq.gz	3729276
43	793_M_case.2.fq.gz	3702132
44	793_M_case.1.fq.gz	3702132
45	1127_M_case.2.fq.gz	3687407
46	1127_M_case.1.fq.gz	3687407
47	55K_F_control.2.fq.gz	3625686
48	55K_F_control.1.fq.gz	3625686
49	44K_M_case.2.fq.gz	3595764
50	44K_M_case.1.fq.gz	3595764
51	64_M_case.2.fq.gz	3595383
52	64_M_case.1.fq.gz	3595383
53	48K_M_case.2.fq.gz	3593399
54	48K_M_case.1.fq.gz	3593399
55	336_M_case.2.fq.gz	3574919
56	336_M_case.1.fq.gz	3574919
57	400_M_case.2.fq.gz	3565863
58	400_M_case.1.fq.gz	3565863
59	1131_M_case.2.fq.gz	3535008
60	1131_M_case.1.fq.gz	3535008
61	402_M_case.2.fq.gz	3526195
62	402_M_case.1.fq.gz	3526195

63	1050_M_control.2.fq.gz	3499077
64	1050_M_control.1.fq.gz	3499077
65	1189_M_control.2.fq.gz	3417931
66	1189_M_control.1.fq.gz	3417931
67	169R_M_case.2.fq.gz	3416127
68	169R_M_case.1.fq.gz	3416127
69	685_M_case.2.fq.gz	3401510
70	685_M_case.1.fq.gz	3401510
71	546_F_control.2.fq.gz	3313990
72	546_F_control.1.fq.gz	3313990
73	1082_M_control.2.fq.gz	3303872
74	1082_M_control.1.fq.gz	3303872
75	835_M_control.2.fq.gz	3294280
76	835_M_control.1.fq.gz	3294280
77	1140_F_control.2.fq.gz	3245711
78	1140_F_control.1.fq.gz	3245711
79	477_M_case.2.fq.gz	3230883
80	477_M_case.1.fq.gz	3230883
81	1338_M_control.2.fq.gz	3200434
82	1338_M_control.1.fq.gz	3200434
83	297_F_control.2.fq.gz	3196413
84	297_F_control.1.fq.gz	3196413
85	1230_F_control.2.fq.gz	3183624
86	1230_F_control.1.fq.gz	3183624
87	894_M_case.2.fq.gz	3175611
88	894_M_case.1.fq.gz	3175611
89	1156_M_case.2.fq.gz	3155945
90	1156_M_case.1.fq.gz	3155945
91	1051_M_control.2.fq.gz	3099024
92	1051_M_control.1.fq.gz	3099024
93	240_M_case.2.fq.gz	3097186
94	240_M_case.1.fq.gz	3097186

95	1195_M_control.2.fq.gz	3095842
96	1195_M_control.1.fq.gz	3095842
97	61_M_control.2.fq.gz	3093468
98	61_M_control.1.fq.gz	3093468
99	52K_M_case.2.fq.gz	3077750
100	52K_M_case.1.fq.gz	3077750
101	1233_M_control.2.fq.gz	3054684
102	1233_M_control.1.fq.gz	3054684
103	5K_M_control.2.fq.gz	2992595
104	5K_M_control.1.fq.gz	2992595
105	1039_M_case.2.fq.gz	2984405
106	1039_M_case.1.fq.gz	2984405
107	534_M_control.2.fq.gz	2961982
108	534_M_control.1.fq.gz	2961982
109	380_M_case.2.fq.gz	2961061
110	380_M_case.1.fq.gz	2961061
111	1227_F_control.2.fq.gz	2958762
112	1227_F_control.1.fq.gz	2958762
113	520_M_case.2.fq.gz	2953103
114	520_M_case.1.fq.gz	2953103
115	26K_M_control.2.fq.gz	2948434
116	26K_M_control.1.fq.gz	2948434
117	43K_M_case.2.fq.gz	2941969
118	43K_M_case.1.fq.gz	2941969
119	347_F_control.2.fq.gz	2934026
120	347_F_control.1.fq.gz	2934026
121	56K_M_case.2.fq.gz	2899619
122	56K_M_case.1.fq.gz	2899619
123	148R_M_control.2.fq.gz	2875846
124	148R_M_control.1.fq.gz	2875846
125	121R_M_case.2.fq.gz	2851913
126	121R_M_case.1.fq.gz	2851913

127	509_M_case.2.fq.gz	2824109
128	509_M_case.1.fq.gz	2824109
129	924_M_case.2.fq.gz	2816594
130	924_M_case.1.fq.gz	2816594
131	1129_M_control.2.fq.gz	2803224
132	1129_M_control.1.fq.gz	2803224
133	22K_M_control.2.fq.gz	2775005
134	22K_M_control.1.fq.gz	2775005
135	1146_M_case.2.fq.gz	2752286
136	1146_M_case.1.fq.gz	2752286
137	505_M_case.2.fq.gz	2752272
138	505_M_case.1.fq.gz	2752272
139	24K_M_control.2.fq.gz	2738075
140	24K_M_control.1.fq.gz	2738075
141	1134_F_case.2.fq.gz	2724490
142	1134_F_case.1.fq.gz	2724490
143	154R_M_case.2.fq.gz	2723171
144	154R_M_case.1.fq.gz	2723171
145	323_M_case.2.fq.gz	2717872
146	323_M_case.1.fq.gz	2717872
147	76R_M_case.2.fq.gz	2699385
148	76R_M_case.1.fq.gz	2699385
149	54K_F_control.2.fq.gz	2687494
150	54K_F_control.1.fq.gz	2687494
151	76_F_control.2.fq.gz	2672243
152	76_F_control.1.fq.gz	2672243
153	804_M_case.2.fq.gz	2654355
154	804_M_case.1.fq.gz	2654355
155	906_M_control.2.fq.gz	2647336
156	906_M_control.1.fq.gz	2647336
157	150R_M_control.2.fq.gz	2624758
158	150R_M_control.1.fq.gz	2624758

159	1222_F_case.2.fq.gz	2616989
160	1222_F_case.1.fq.gz	2616989
161	379_M_case.2.fq.gz	2613759
162	379_M_case.1.fq.gz	2613759
163	56_M_case.2.fq.gz	2613162
164	56_M_case.1.fq.gz	2613162
165	373_M_case.2.fq.gz	2610532
166	373_M_case.1.fq.gz	2610532
167	1052_F_control.2.fq.gz	2608208
168	1052_F_control.1.fq.gz	2608208
169	994_M_case.2.fq.gz	2603899
170	994_M_case.1.fq.gz	2603899
171	721_F_control.2.fq.gz	2601545
172	721_F_control.1.fq.gz	2601545
173	392_M_case.2.fq.gz	2601021
174	392_M_case.1.fq.gz	2601021
175	1153_F_control.2.fq.gz	2600040
176	1153_F_control.1.fq.gz	2600040
177	512_M_case.2.fq.gz	2580102
178	512_M_case.1.fq.gz	2580102
179	1231_M_control.2.fq.gz	2562660
180	1231_M_control.1.fq.gz	2562660
181	849_M_case.2.fq.gz	2555807
182	849_M_case.1.fq.gz	2555807
183	1312_M_control.2.fq.gz	2460239
184	1312_M_control.1.fq.gz	2460239
185	642_M_case.2.fq.gz	2427540
186	642_M_case.1.fq.gz	2427540
187	303=518_M_case.2.fq.gz	2414062
188	303=518_M_case.1.fq.gz	2414062
189	63K_F_control.2.fq.gz	2388074
190	63K_F_control.1.fq.gz	2388074

191	517_M_case.2.fq.gz	2355408
192	517_M_case.1.fq.gz	2355408
193	407_M_control.2.fq.gz	2344138
194	407_M_control.1.fq.gz	2344138
195	1037_M_case.2.fq.gz	2342117
196	1037_M_case.1.fq.gz	2342117
197	1022_M_case.2.fq.gz	2329217
198	1022_M_case.1.fq.gz	2329217
199	83R_M_case.2.fq.gz	2308326
200	83R_M_case.1.fq.gz	2308326
201	478_M_case.2.fq.gz	2300692
202	478_M_case.1.fq.gz	2300692
203	79R_M_control.2.fq.gz	2294643
204	79R_M_control.1.fq.gz	2294643
205	1009_M_case.2.fq.gz	2292534
206	1009_M_case.1.fq.gz	2292534
207	862_F_case.2.fq.gz	2287408
208	862_F_case.1.fq.gz	2287408
209	792_M_control.2.fq.gz	2277217
210	792_M_control.1.fq.gz	2277217
211	142R_F_control.2.fq.gz	2232527
212	142R_F_control.1.fq.gz	2232527
213	1193_M_case.2.fq.gz	2199946
214	1193_M_case.1.fq.gz	2199946
215	1157_M_case.2.fq.gz	2179797
216	1157_M_case.1.fq.gz	2179797
217	1232_M_control.2.fq.gz	2176808
218	1232_M_control.1.fq.gz	2176808
219	1017_M_case.2.fq.gz	2166669
220	1017_M_case.1.fq.gz	2166669
221	1137_M_case.2.fq.gz	2164521
222	1137_M_case.1.fq.gz	2164521

223	939_M_case.2.fq.gz	2159613
224	939_M_case.1.fq.gz	2159613
225	469_M_case.2.fq.gz	2148210
226	469_M_case.1.fq.gz	2148210
227	1047_M_control.2.fq.gz	2139338
228	1047_M_control.1.fq.gz	2139338
229	828_M_case.2.fq.gz	2098709
230	828_M_case.1.fq.gz	2098709
231	140R_M_case.2.fq.gz	2095201
232	140R_M_case.1.fq.gz	2095201
233	827_M_case.2.fq.gz	2088569
234	827_M_case.1.fq.gz	2088569
235	516_M_case.2.fq.gz	2079830
236	516_M_case.1.fq.gz	2079830
237	1303_M_control.2.fq.gz	2077683
238	1303_M_control.1.fq.gz	2077683
239	397_M_case.2.fq.gz	2061828
240	397_M_case.1.fq.gz	2061828
241	456_M_case.2.fq.gz	2042228
242	456_M_case.1.fq.gz	2042228
243	84R_M_case.2.fq.gz	2036099
244	84R_M_case.1.fq.gz	2036099
245	1185_F_control.2.fq.gz	2015275
246	1185_F_control.1.fq.gz	2015275
247	503_M_case.2.fq.gz	1980700
248	503_M_case.1.fq.gz	1980700
249	1277_M_control.2.fq.gz	1978178
250	1277_M_control.1.fq.gz	1978178
251	46K_M_case.2.fq.gz	1974958
252	46K_M_case.1.fq.gz	1974958
253	25K_M_control.2.fq.gz	1959995
254	25K_M_control.1.fq.gz	1959995

255	1304_M_control.2.fq.gz	1958898
256	1304_M_control.1.fq.gz	1958898
257	23K_M_control.2.fq.gz	1939822
258	23K_M_control.1.fq.gz	1939822
259	227_M_case.2.fq.gz	1923671
260	227_M_case.1.fq.gz	1923671
261	826_M_control.2.fq.gz	1922173
262	826_M_control.1.fq.gz	1922173
263	1026_M_case.2.fq.gz	1916052
264	1026_M_case.1.fq.gz	1916052
265	1155_F_control.2.fq.gz	1897867
266	1155_F_control.1.fq.gz	1897867
267	672_M_case.2.fq.gz	1890123
268	672_M_case.1.fq.gz	1890123
269	720_M_case.2.fq.gz	1873564
270	720_M_case.1.fq.gz	1873564
271	30K_F_case.2.fq.gz	1853575
272	30K_F_case.1.fq.gz	1853575
273	4K_F_control.2.fq.gz	1840936
274	4K_F_control.1.fq.gz	1840936
275	1033_M_case.2.fq.gz	1833029
276	1033_M_case.1.fq.gz	1833029
277	372_M_case.2.fq.gz	1827428
278	372_M_case.1.fq.gz	1827428
279	726_M_control.2.fq.gz	1824749
280	726_M_control.1.fq.gz	1824749
281	593_M_control.2.fq.gz	1814251
282	593_M_control.1.fq.gz	1814251
283	910_M_case.2.fq.gz	1804819
284	910_M_case.1.fq.gz	1804819
285	1184_M_control.2.fq.gz	1798938
286	1184_M_control.1.fq.gz	1798938

287	1013_F_case.2.fq.gz	1763845
288	1013_F_case.1.fq.gz	1763845
289	1014_F_case.2.fq.gz	1759027
290	1014_F_case.1.fq.gz	1759027
291	28K_M_control.2.fq.gz	1751856
292	28K_M_control.1.fq.gz	1751856
293	797_F_control.2.fq.gz	1750878
294	797_F_control.1.fq.gz	1750878
295	99R_M_case.2.fq.gz	1748394
296	99R_M_case.1.fq.gz	1748394
297	1152_M_control.2.fq.gz	1665912
298	1152_M_control.1.fq.gz	1665912
299	1188_F_control.2.fq.gz	1657469
300	1188_F_control.1.fq.gz	1657469
301	933_M_control.2.fq.gz	1649478
302	933_M_control.1.fq.gz	1649478
303	913_M_case.2.fq.gz	1643078
304	913_M_case.1.fq.gz	1643078
305	905_M_case.2.fq.gz	1633370
306	905_M_case.1.fq.gz	1633370
307	919_M_case.2.fq.gz	1620840
308	919_M_case.1.fq.gz	1620840
309	77R_M_case.2.fq.gz	1619510
310	77R_M_case.1.fq.gz	1619510
311	1151_F_control.2.fq.gz	1612265
312	1151_F_control.1.fq.gz	1612265
313	36K_M_case.2.fq.gz	1607359
314	36K_M_case.1.fq.gz	1607359
315	78R_M_case.2.fq.gz	1601575
316	78R_M_case.1.fq.gz	1601575
317	653_M_case.2.fq.gz	1600696
318	653_M_case.1.fq.gz	1600696

319	1194_F_case.2.fq.gz	1596143
320	1194_F_case.1.fq.gz	1596143
321	912_M_case.2.fq.gz	1595837
322	912_M_case.1.fq.gz	1595837
323	508_M_case.2.fq.gz	1592693
324	508_M_case.1.fq.gz	1592693
325	321_M_case.2.fq.gz	1590764
326	321_M_case.1.fq.gz	1590764
327	16K_F_case.2.fq.gz	1580755
328	16K_F_case.1.fq.gz	1580755
329	62_M_case.2.fq.gz	1580126
330	62_M_case.1.fq.gz	1580126
331	62K_F_control.2.fq.gz	1574165
332	62K_F_control.1.fq.gz	1574165
333	295=519_M_control.2.fq.gz	1560190
334	295=519_M_control.1.fq.gz	1560190
335	27K_M_control.2.fq.gz	1544751
336	27K_M_control.1.fq.gz	1544751
337	1073_M_control.2.fq.gz	1538948
338	1073_M_control.1.fq.gz	1538948
339	334_M_case.2.fq.gz	1485702
340	334_M_case.1.fq.gz	1485702
341	387_M_case.2.fq.gz	1482658
342	387_M_case.1.fq.gz	1482658
343	29K_M_case.2.fq.gz	1464272
344	29K_M_case.1.fq.gz	1464272
345	1187_F_control.2.fq.gz	1444808
346	1187_F_control.1.fq.gz	1444808
347	836_M_case.2.fq.gz	1422793
348	836_M_case.1.fq.gz	1422793
349	361_M_case.2.fq.gz	1404417
350	361_M_case.1.fq.gz	1404417

351	812_M_control.2.fq.gz	1403228
352	812_M_control.1.fq.gz	1403228
353	103R_M_control.2.fq.gz	1399253
354	103R_M_control.1.fq.gz	1399253
355	501_M_case.2.fq.gz	1361901
356	501_M_case.1.fq.gz	1361901
357	1170_M_case.2.fq.gz	1341014
358	1170_M_case.1.fq.gz	1341014
359	438_M_case.2.fq.gz	1338109
360	438_M_case.1.fq.gz	1338109
361	522_M_control.2.fq.gz	1332571
362	522_M_control.1.fq.gz	1332571
363	1088_M_case.2.fq.gz	1330903
364	1088_M_case.1.fq.gz	1330903
365	151R_M_control.2.fq.gz	1322416
366	151R_M_control.1.fq.gz	1322416
367	911_M_case.2.fq.gz	1317165
368	911_M_case.1.fq.gz	1317165
369	375_M_case.2.fq.gz	1301986
370	375_M_case.1.fq.gz	1301986
371	480_M_case.2.fq.gz	1299314
372	480_M_case.1.fq.gz	1299314
373	187R_M_control.2.fq.gz	1281581
374	187R_M_control.1.fq.gz	1281581
375	66K_F_case.2.fq.gz	1270243
376	66K_F_case.1.fq.gz	1270243
377	643_M_case.2.fq.gz	1267039
378	643_M_case.1.fq.gz	1267039
379	408_M_case.2.fq.gz	1263469
380	408_M_case.1.fq.gz	1263469
381	652_M_case.2.fq.gz	1256588
382	652_M_case.1.fq.gz	1256588

383	1266_F_control.2.fq.gz	1240596
384	1266_F_control.1.fq.gz	1240596
385	1217_M_case.2.fq.gz	1239214
386	1217_M_case.1.fq.gz	1239214
387	915_M_case.2.fq.gz	1234941
388	915_M_case.1.fq.gz	1234941
389	904_M_case.2.fq.gz	1205645
390	904_M_case.1.fq.gz	1205645
391	742_M_control.2.fq.gz	1192490
392	742_M_control.1.fq.gz	1192490
393	532_M_case.2.fq.gz	1185117
394	532_M_case.1.fq.gz	1185117
395	144R_M_control.2.fq.gz	1182816
396	144R_M_control.1.fq.gz	1182816
397	69_M_case.2.fq.gz	1175933
398	69_M_case.1.fq.gz	1175933
399	2K_M_case.2.fq.gz	1175375
400	2K_M_case.1.fq.gz	1175375
401	340_F_control.2.fq.gz	1158391
402	340_F_control.1.fq.gz	1158391
403	993_M_control.2.fq.gz	1144414
404	993_M_control.1.fq.gz	1144414
405	1149_M_case.2.fq.gz	1133392
406	1149_M_case.1.fq.gz	1133392
407	12K_F_control.2.fq.gz	1108165
408	12K_F_control.1.fq.gz	1108165
409	521_M_control.2.fq.gz	1105142
410	521_M_control.1.fq.gz	1105142
411	21K_M_control.2.fq.gz	1073301
412	21K_M_control.1.fq.gz	1073301
413	319_M_case.2.fq.gz	1049122
414	319_M_case.1.fq.gz	1049122

415	472_M_case.2.fq.gz	1047467
416	472_M_case.1.fq.gz	1047467
417	337_F_control.2.fq.gz	1034769
418	337_F_control.1.fq.gz	1034769
419	920_M_case.2.fq.gz	1014384
420	920_M_case.1.fq.gz	1014384
421	1148_M_control.2.fq.gz	995468
422	1148_M_control.1.fq.gz	995468
423	45K_M_control.2.fq.gz	982599
424	45K_M_control.1.fq.gz	982599
425	918_M_case.2.fq.gz	971382
426	918_M_case.1.fq.gz	971382
427	695_M_case.2.fq.gz	945398
428	695_M_case.1.fq.gz	945398
429	601_M_case.2.fq.gz	933862
430	601_M_case.1.fq.gz	933862
431	1311_M_case.2.fq.gz	930548
432	1311_M_case.1.fq.gz	930548
433	641_M_case.2.fq.gz	914931
434	641_M_case.1.fq.gz	914931
435	61K_F_case.2.fq.gz	905976
436	61K_F_case.1.fq.gz	905976
437	514_M_case.2.fq.gz	902155
438	514_M_case.1.fq.gz	902155
439	14K_F_control.2.fq.gz	897889
440	14K_F_control.1.fq.gz	897889
441	331_M_control.2.fq.gz	888838
442	331_M_control.1.fq.gz	888838
443	1141_M_case.2.fq.gz	886779
444	1141_M_case.1.fq.gz	886779
445	410_M_case.2.fq.gz	869204
446	410_M_case.1.fq.gz	869204

447	3K_M_control.2.fq.gz	850483
448	3K_M_control.1.fq.gz	850483
449	1048_M_control.2.fq.gz	830430
450	1048_M_control.1.fq.gz	830430
451	430_M_case.2.fq.gz	822470
452	430_M_case.1.fq.gz	822470
453	339_M_case.2.fq.gz	819419
454	339_M_case.1.fq.gz	819419
455	42K_M_case.2.fq.gz	808304
456	42K_M_case.1.fq.gz	808304
457	63_M_control.2.fq.gz	803234
458	63_M_control.1.fq.gz	803234
459	312_M_case.2.fq.gz	788798
460	312_M_case.1.fq.gz	788798
461	64K_M_control.2.fq.gz	786608
462	64K_M_control.1.fq.gz	786608
463	817_M_case.2.fq.gz	785583
464	817_M_case.1.fq.gz	785583
465	724_F_control.2.fq.gz	771171
466	724_F_control.1.fq.gz	771171
467	1229_F_control.2.fq.gz	759211
468	1229_F_control.1.fq.gz	759211
469	1299_F_control.2.fq.gz	693971
470	1299_F_control.1.fq.gz	693971
471	454_M_case.2.fq.gz	693662
472	454_M_case.1.fq.gz	693662
473	934_M_control.2.fq.gz	688899
474	934_M_control.1.fq.gz	688899
475	1136_M_case.2.fq.gz	673780
476	1136_M_case.1.fq.gz	673780
477	762_M_case.2.fq.gz	667399
478	762_M_case.1.fq.gz	667399

479	405_F_control.2.fq.gz	664639
480	405_F_control.1.fq.gz	664639
481	907_F_case.2.fq.gz	658437
482	907_F_case.1.fq.gz	658437
483	659_M_case.2.fq.gz	631821
484	659_M_case.1.fq.gz	631821
485	723_F_control.2.fq.gz	620856
486	723_F_control.1.fq.gz	620856
487	722_M_control.2.fq.gz	617601
488	722_M_control.1.fq.gz	617601
489	101R_M_case.2.fq.gz	605527
490	101R_M_case.1.fq.gz	605527
491	1126_M_control.2.fq.gz	604265
492	1126_M_control.1.fq.gz	604265
493	119R_M_case.2.fq.gz	594255
494	119R_M_case.1.fq.gz	594255
495	188R_M_control.2.fq.gz	592753
496	188R_M_control.1.fq.gz	592753
497	1150_M_case.2.fq.gz	539295
498	1150_M_case.1.fq.gz	539295
499	1040_M_case.2.fq.gz	536748
500	1040_M_case.1.fq.gz	536748
501	70_M_control.2.fq.gz	528327
502	70_M_control.1.fq.gz	528327
503	543_F_control.2.fq.gz	475306
504	543_F_control.1.fq.gz	475306
505	507_M_case.2.fq.gz	460840
506	507_M_case.1.fq.gz	460840
507	834_M_case.2.fq.gz	419587
508	834_M_case.1.fq.gz	419587
509	536_M_case.2.fq.gz	370978
510	536_M_case.1.fq.gz	370978

511	1016_M_case.2.fq.gz	295738
512	1016_M_case.1.fq.gz	295738
	Total	1075960322
	min	295738
	max	6333140
	average	2101485.004
	Stddev	1099483.93258753
	median	1949360

S1 Text. Command lines for *de novo, Bos Taurus* reference and European bison reference pipeline, with and without PCR duplicates

1. Commands used for *de novo* parameter optimization

(i) denovo_map.pl -M 2 -n 2 -T 8 --samples ./samples/ --popmap ./popmaps/popmap.tsv -o ./M2/ --paired -r 0.80;
(ii) denovo_map.pl -M 3 -n 3 -T 8 --samples ./samples/ --popmap ./popmaps/popmap.tsv -o ./M3/ --paired -r 0.80;
(iii) denovo_map.pl -M 4 -n 4 -T 8 --samples ./samples/ --popmap ./popmaps/popmap.tsv -o ./M4/ --paired -r 0.80;
(iv) denovo_map.pl -M 5 -n 5 -T 8 --samples ./samples/ --popmap ./popmaps/popmap.tsv -o ./M5/ --paired -r 0.80;
(v) denovo_map.pl -M 6 -n 6 -T 8 --samples ./samples/ --popmap ./popmaps/popmap.tsv -o ./M6/ --paired -r 0.80;
(vi) denovo_map.pl -M 7 -n 7 -T 8 --samples ./samples/ --popmap ./popmaps/popmap.tsv -o ./M7/ --paired -r 0.80

After Denovo, we have calculated the increasing number of loci in respective folder, M2, M3, M4, M5, M6, M7 by using line count (wc –l)

(i) wc -l M2/populations.hapstats.tsv 7222
(ii) wc -l M3/populations.hapstats.tsv 7094
(iii) wc -l M4/populations.hapstats.tsv 7087
(iv) wc -l M5/populations.hapstats.tsv 7152
(v) wc -l M6/populations.hapstats.tsv 7169
(vi) wc -l M7/populations.hapstats.tsv 7207

###To create r80_loci.tsv

(i)	M2/M3	M3-M2	7094-7222=-128
(ii)	M3/M4	M4-M3	7087-7094=-7
(iii)	M4/M5	M5-M4	7152-7087=65
(iv)	M5/M6	M6-M5	7169-7152=17
(v)	M6/M7	M7-M6	7207-7169=38

Graph has been plotted using r80_loci.tsv file in GNUPLOT (version 5.2, http://www.gnuplot.info/).

2. *De novo* pipeline with and without PCR duplicates commands

   (i) denovo_map.pl -M 4 -n 4 -T 8 --samples ./fastq_files/ --popmap ./popmaps/popmap.tsv -o ./denovo_pcr/ --paired -r 0.80

   (ii) denovo_map.pl -M 4 -n 4 -T 8 --samples ./fastq_files/ --popmap ./popmaps/popmap.tsv -o ./denovo_rmpcr/ --paired -r 0.80 --rm-pcr-duplicates

   (iii) populations -P . --popmap popmap.tsv --vcf -t 8 -O Denovo_pcr/rmpcr_r0.80_vcf/ --min-samples-per-pop 0.80

   (iv) vcftools --vcf populations.snps.vcf --maf 0.05 --minGQ 15 --max-missing 0.5 --out filtered_SNP --recode

3. *Bos taurus* reference with and without PCR duplicates commands

   (i) bwa mem -t 8 ./UMD3.1/BWAIndex/version0.6.0/genome.fa sample_name.1.fq.gz sample_name.2.fq.gz | samtools view -h -b | samtools sort --threads 2 > sample_name_sorted.bam

   (ii) ref_map.pl -T 8 -o ./UMD3.1_pcr_r0.80/ --popmap ./popmap.tsv --samples ./UMD3.1_BWA_sorted_bam/ -X populations: --min-samples-per-pop 0.80 --vcf --ordered-export

   (iii) ref_map.pl -T 8 -o ./UMD3.1_rmpcr_r0.80/ --popmap ./popmap.tsv --samples ./UMD3.1_BWA_sorted_bam/ --rm-pcr-duplicates -X populations: --min-samples-per-pop 0.80 --vcf --ordered-export

   (iv) vcftools --vcf populations.snps.vcf --maf 0.05 --minGQ 15 --max-missing 0.5 --out filtered_SNP –recode

(v) grep -v "^#" filtered_populations.snps.vcf | awk -F'\t' '{print $1 "\t" $2}' | sort -n -k2 > *Bostaurus*_reference_populations.snps.vcf_chr_pos_sort.txt

(vi)

4. European bison reference with and without PCR duplicates commands

(vii) bwa mem -t 8 ./EuropeanBison/BWAIndex/wisent.fa sample_name.1.fq.gz sample_name.2.fq.gz | samtools view -h -b | samtools sort --threads 2 > sample_name_sorted.bam

(viii) ref_map.pl -T 8 -o ./ EuropeanBison_pcr_r0.80/ --popmap ./popmap.tsv --samples ./EuropeanBison_BWA_sorted_bam/ -X populations: --min-samples-per-pop 0.80 --vcf --ordered-export

(ix) ref_map.pl -T 8 -o ./ EuropeanBison_rmpcr_r0.80/ --popmap ./popmap.tsv --samples ./UMD3.1_BWA_sorted_bam/ --rm-pcr-duplicates -X populations: --min-samples-per-pop 0.80 --vcf --ordered-export

(x) vcftools --vcf populations.snps.vcf --maf 0.05 --minGQ 15 --max-missing 0.5 --out filtered_SNP --recode

5. *denovo/Bos taurus* and *de novo*/European bison, with and without PCR duplicates commands

(i) bwa mem -t 8 ./UMD3.1/BWAIndex/version0.6.0/genome.fa ./denovo_pcr/catalog.fa.gz | samtools view -b | samtools sort > Denovo/Bostaurus_pcr.bam

(ii) bwa mem -t 8 ./UMD3.1/BWAIndex/version0.6.0/genome.fa ./denovo_rmpcr/catalog.fa.gz | samtools view -b | samtools sort > Denovo/Bostaurus_rmpcr.bam

(iii) stacks-integrate-alignments -P . -B Denovo/Bostaurus_rmpcr.bam -O Denovo/Bostaurus_rmpcr_population/

(iv) stacks-integrate-alignments -P . -B Denovo/Bostaurus_pcr.bam -O Denovo/Bostaurus_pcr_population/

(v) populations -P . --popmap popmap.tsv --vcf -t 8 -O Denovo/Bostaurus_pcr_population/ --min-samples-per-pop 0.80

(vi) vcftools --vcf populations.snps.vcf --maf 0.05 --minGQ 15 --max-missing 0.5 --out filtered_SNP –recode

(vii) grep -v "^#" populations.snps.vcf | awk -F'\t' '{print $1 "\t" $2}' | sort -n -k2 > *denovo/Bos taurus*_populations.snps.vcf_chr_pos_sort.txt

(xi) awk 'NR==FNR { array[$0]; next } $0 in array' *denovo/Bos taurus*_populations.snps.vcf_chr_pos_sort.txt *Bostaurus*_reference_populations.snps.vcf_chr_pos_sort.txt

Grep and Awk Commands:-

1. To extract only two columns from VCF file

```
grep -v "^#"  file_name.vcf | awk -F'\t' '{print $1 "\t" $2}' | sort -n -k2 >
file_name.vcf_chr_pos_sort.txt
```

2. To check common variant from two files

```
awk 'NR==FNR { array[$0]; next } $0 in array'  file_name.vcf_chr_pos_sort.txt
file_name.vcf_chr_pos_sort.txt
```

Table S3. Summary genetics and statistics calculated by the *Stacks* population program for variant (Polymorphic) loci and all loci using two different approaches: reference-based (*Bos Taurus* (*B. taurus*) and European bison (E. bison)) and *de novo,* all with and without PCR duplicates (PCR dupl. and no PCR dupl.).

	***B. taurus* reference:**		**E. bison reference:**		***de novo***	
	**PCR dupl.**	**no PCR dupl.**	**PCR dupl.**	**no PCR dupl.**	**PCR dupl.**	**no PCR dupl.**
			**All loci**			
Sites	426948	353847	547686	456391	135002	70361
Variant_Sites	2452	3110	2970	4848	681	980
Poly.Sites	2452	3110	2970	4848	681	980
%Poly.Loci	0.57431	0.87891	0.54228	1.06225	0.50444	1.39282
Num_Indv	219.3172	219.3517	220.9424	220.8788	228.6148	219.9141
P	0.99986	0.99958	0.99986	0.99958	0.9996	0.99969
Obs_Het	0.00028	0.00047	0.00029	0.00048	0.00074	0.00061
Obs_Hom	0.99972	0.99953	0.99971	0.99952	0.99926	0.99939
Exp_Het	0.00017	0.00065	0.00017	0.00068	0.00047	0.00055
Fis	-0.00012	0.00174	-0.00012	0.00195	-0.00027	-0.00012
Var	0.00031	0.00226	0.00034	0.00241	0.00089	0.00029
StdErr	0.01999	0.022	0.01857	0.02042	0.04156	0.03731
			**Variant loci**			
Num_Indv	212.7525	219.2949	213.3562	217.9899	222.0308	216.8439
P	0.97567	0.95232	0.97338	0.9606	0.92118	0.97801
Obs_Het	0.04842	0.05308	0.0529	0.04529	0.14608	0.04369
Obs_Hom	0.95158	0.94692	0.9471	0.95471	0.85392	0.95631
Exp_Het	0.02969	0.0737	0.03146	0.06429	0.0922	0.03961
Fis	-0.02122	0.19808	-0.02303	0.18402	-0.05428	-0.00896
Var	0.05327	0.21865	0.06244	0.19384	0.17291	0.02092
StdErr	0.15438	0.20512	0.15109	0.15275	0.49407	0.28534

Num_Indv - average number of individuals at each locus; % Poly. Loci – percentage of polymorphic loci found polymorphic within the population; P – average frequency of the major allele at each locus in this population; Obs_Het – average observed heterozygosity per locus; ExpHet – expected heterozygosity; Obs_Hom – average observed homozygosity per locus; FIS – average FIS estimations across loci.
